# COVID-19 Impact on Health Behaviors and Survey Engagement in Older African Americans: Longitudinal Cohort Study

**DOI:** 10.2196/79432

**Published:** 2026-08-03

**Authors:** Selda Yildiz, Nora Mattek, Joel Steele, Sarah Gothard, Bryan D James, Ana W Capuano, Lisa L Barnes, Jeffrey A Kaye, Zachary T Beattie

**Affiliations:** 1 Department of Neurology Oregon Health & Science University Portland, OR United States; 2 Layton Aging & Alzheimer's Disease Center Oregon Health & Science University Portland, OR United States; 3 Oregon Center for Aging & Technology (ORCATECH) Oregon Health & Science University Portland, OR United States; 4 Department of Indigenous Health University of North Dakota Grand Forks, ND United States; 5 Department of Epidemiology University of Washington Seattle, WA United States; 6 Rush Alzheimer's Disease Center Rush University Medical Center Chicago, IL United States; 7 Department of Internal Medicine Rush University Medical Center Chicago, IL United States; 8 Department of Neurology University of Chicago Chicago, IL United States; 9 Department of Public Health Sciences University of Chicago Chicago, IL United States

**Keywords:** actigraphy, aging, COVID-19, digital technology, health disparities, MCI, mild cognitive impairment, older adults, online surveys, physical activity, remote monitoring, sleep, wearables

## Abstract

**Background:**

The COVID-19 pandemic disrupted older adults’ daily lives, particularly concerning social interaction, physical activity, and sleep quality. Older African Americans were disproportionately affected yet remain underrepresented in research documenting the impact of the COVID-19 pandemic.

**Objective:**

This study investigated changes in self-reported health, survey engagement, physical activity, and sleep duration among older African American adults in the Minority Aging Research Study (MARS) before and after Illinois’ March 21, 2020, COVID-19 stay-at-home order, using online surveys and actigraphy watch data.

**Methods:**

MARS is a longitudinal observational cohort study of older African American adults who enroll initially without dementia. We examined a subset of MARS participants enrolled in the Collaborative Aging Research Using Technology initiative. Weekly online health survey responses to binary (yes/no) questions (eg, away from home overnight, overnight visitors, blue mood, loneliness, medication changes, falls, accidents, hospitalizations, health limitations, living space change, or assistance change) were analyzed for 32 weeks (November 30, 2019, to July 11, 2020) and actigraphy data for over 10 weeks (February 15, 2020, to April 15, 2020). Generalized linear mixed models with a logit link function for binary outcomes and linear mixed models for continuous outcomes, adjusted for age, sex, and education, were used to assess changes in self-reported experiences and actigraphy-derived daily steps and nightly sleep and reported as odds ratios (ORs) with 95% credible intervals (CrIs).

**Results:**

Of 59 participants (mean age 76.6, SD 6.1 years; male: 11/59, 19%) included in the survey data analysis, 43 (73%) were classified as high-engagement (completed at least 50% of the weekly surveys) and 16 (27%) as low-engagement; these participants were more likely to have mild cognitive impairment (3/43, 7% vs 5/16, 31%; *P*=.03) and lower mean Mini-Mental State Examination scores (28.1, SD 1.4 vs 28.9, SD 1.0; *P*=.04). Generalized linear mixed models on the full analytic sample (N=59) showed significant reductions post–COVID-19 in being away from home overnight (OR 0.30, 95% CrI 0.20-0.46), having overnight visitors (OR 0.44, 95% CrI 0.31-0.64), a medication change (OR 0.60, 95% CrI 0.42-0.86), and a health limitation (OR 0.70, 95% CrI 0.52-0.95). COVID-19 and study-related technical disruptions limited actigraphy data availability. Among 15 participants with valid data, mean daily step count decreased significantly (20.1%; 1646, SD 1306 to 1315, SD 1149 steps; *P*<.001); nightly sleep duration decreased but not significantly (2.8%; 7.1, SD 2.3 hours to 6.9, SD 2.3 hours; *P*=.49).

**Conclusions:**

Despite widespread COVID-19 disruptions, older African American MARS participants maintained stable survey engagement. Participants with low survey engagement were more likely to have cognitive impairment, suggesting that mild cognitive challenges may hinder sustained participation with online responses. A subset with valid actigraphy data showed reduced physical activity. Although technical issues limited data availability, findings support the value of objective monitoring and highlight the challenges associated with public health disruptions to research infrastructure.

## Introduction

The COVID-19 pandemic significantly impacted the well-being of older adults, particularly concerning social isolation, loneliness, hospitalization, medication changes, physical activity, and sleep quality, among others [1-6], while disproportionately affecting older African American adults [7-9]. Increased social isolation, loneliness, anxiety, depression, poor sleep quality, and reduced physical activity were common among older adults during the COVID-19 pandemic due to public health restrictions and social distancing mandates [1,2,6,10].

Recent longitudinal studies using information and communication technology and/or digital health technologies have provided unique opportunities to examine pre– and post–COVID-19 pandemic behavioral and social patterns among older adults [11-15]. For instance, in The Irish Longitudinal Study on Ageing, Briggs et al [11] reported an increase in the burden of depressive symptoms among older adults, particularly those living alone, during the COVID-19 pandemic. Similarly, the Internet-Based Conversational Engagement Clinical Trial previously reported decreased social interaction time among older adults during the COVID-19 pandemic [13]. In addition, changes in the daily behaviors of older adults were identified during the early phases of the COVID-19 pandemic, with reductions in daily driving and increased loneliness and blue mood [12]. Similarly, a study of rural and urban veterans and their cohabitants found that the COVID-19 pandemic was associated with significant changes in mood and physical activity, underscoring the widespread behavioral health impact of pandemic-related disruptions across diverse populations [14].

These patterns are particularly concerning given that social connectedness is a well-documented protective factor for overall well-being and healthy aging [16,17], whereas social isolation and loneliness have been consistently linked to increased health risks, including a greater risk of depression [18], dementia [19], cardiovascular disease [20], and premature mortality [21,22]. Importantly, when compared with other racial and ethnic groups, older African American adults were at higher risk of social isolation and loneliness during the pandemic [8,23]. Data from the 2003-2020 American Time Use Survey revealed more social isolation and less social engagement among African Americans relative to other racial groups [24]. Additionally, across multiple investigations from a dietary lifestyle intervention of African Americans in the Southeastern United States, the Nutritious Eating With Soul study reported that the early phases of the COVID-19 pandemic led to increased stress (regarding nutrition, work, and family), changes in routines, reduced exercise, increased cognitive control, and increased BMI among African Americans [25,26].

Despite these contributions, 2 gaps remain. First, older African Americans remain underrepresented in digital monitoring studies conducted during the COVID-19 pandemic, despite being disproportionately affected by COVID-19. Second, prior research has primarily focused on whether older adults’ health and behavior changed during the pandemic, rather than also exploring whether they were able to sustain engagement with longitudinal research and digital monitoring through it, which is a distinct question with direct implications for the resilience of remote research infrastructure during future public health disruptions.

To address these gaps, we explored a subset of data from a cohort of African American older adults in Chicago within the Minority Aging Research Study (MARS) at Rush University, which integrates the digital health infrastructure from the Oregon Center for Aging and Technology (ORCATECH) Collaborative Aging Research Using Technology (CART) initiative [27,28].

The ORCATECH CART initiative, led by Oregon Health and Science University (OHSU), has previously developed a research infrastructure to use digital health technologies for investigating healthy and independent aging longitudinally in 4 diverse cohorts of older adults across the United States: low-income residents in Portland, Latinx and Hispanic residents in Miami, predominantly rural-residing veterans in the Pacific Northwest, and urban African American older adults in Chicago via the MARS within the Rush Alzheimer’s Disease Center. MARS, funded by the National Institute on Aging, is one of the largest and longest-running longitudinal epidemiologic cohort studies focusing on aging in older African Americans. Its primary objective is to identify individual and environmental race-specific stressors that account for cognitive decline among older African Americans without known dementia at baseline. MARS data collection began in August 2004, and the CART infrastructure was added in 2018 to support digital health monitoring. More in-depth study details for MARS have previously been published [29-31]. MARS provides a unique opportunity to understand the impact of the early phases of COVID-19–mandated stay-at-home orders and restricted in-person interactions on the behavioral and lifestyle patterns of older African American participants.

Within this substudy of MARS, our goals were to (1) assess survey engagement before and after the onset of Illinois’ COVID-19 stay-at-home order on March 21, 2020; (2) analyze changes in a set of behavioral, social, and health patterns using weekly online questionnaires, as well as physical activity and sleep using actigraphy watch data; (3) examine, in post hoc exploratory analyses, whether survey engagement was associated with mild cognitive impairment (MCI); and (4) examine participant interaction with the survey platform, including start-hour timing and survey completion time.

## Methods

### Ethical Considerations

This study was approved by the Institutional Review Boards (IRBs) of OHSU (IRB #23921) and Rush University (IRB #16011407) prior to the recruitment of participants to ensure the ethical conduct of the study. All study participants provided written informed consent prior to all study procedures and, during the consent process, were informed that the research findings would be published in reports, articles, and presentations. All study data were anonymized and deidentified. Participant compensation was US $50 per month per home and was distributed quarterly via a US $150 Visa gift card.

### Study Participants

Study participants were enrolled as part of the MARS at the Rush Alzheimer’s Disease Center, Rush University Medical Center, in Chicago, Illinois. MARS recruitment included churches, subsidized senior housing facilities, retirement communities, African American clubs, organizations, fraternities and sororities, and social service centers serving seniors in metropolitan Chicago and the outlying suburbs [30]. OHSU and ORCATECH in Portland, Oregon, assisted with data management and conducted data analysis. Participants included in this report completed baseline MARS evaluations and were all actively enrolled during the data analysis period between November 30, 2019, and July 11, 2020.

### Study Inclusion and Exclusion Criteria

Inclusion criteria for the full MARS study were self-identified non-Hispanic African Americans who were aged 65 years or older without known dementia. CART initiative enrollment criteria included being aged 62 years or older, living alone or with a spouse/partner in a residence larger than 1 room with a reliable internet connection, having experience with basic computer use (eg, sending and receiving email), willingness and ability to complete weekly online health surveys, and wearing an actigraphy watch continuously, day and night, throughout the entire study period. Although CART’s overall age criterion across its 4 cohorts is being aged 62 years or older, MARS-CART participants must also meet the MARS criterion of being aged 65 years or older; thus, the effective minimum age for participants in this MARS-CART study was 65 years. Exclusion criteria for MARS-CART were inability to provide informed consent, and additionally for CART were inability to physically participate in the study (eg, being a wheelchair user), living with more than 1 person in a residence, and any uncontrolled medical condition that was expected to preclude completion of the study (eg, late-stage cancers). A full list of MARS-CART inclusion and exclusion criteria is provided in Textbox 1.

**Minority Aging Research Study–Collaborative Aging Research Using Technology (MARS-CART) inclusion and exclusion criteria.**

**Inclusion criteria**

- Aged 65 years or older
- Self-identification as non-Hispanic African American
- Without a known dementia diagnosis
- Living alone or with a spouse or partner in a residence larger than 1 room
- Reliable internet connection
- Experience with basic computer use
- Willingness and ability to complete weekly online health surveys and wear an actigraphy watch continuously day and night throughout the entire study period

**Exclusion Criteria**

- Inability to provide informed consent
- Inability to physically participate in the study (eg, being a wheelchair user)
- More than 2 people living in the participant’s residence
- Any uncontrolled medical condition that is expected to preclude completion of the study (eg, late-stage cancers)

### Data Collection

#### Participant Baseline and Annual Evaluations

The full MARS protocol for participant screening includes an initial in-person baseline evaluation comprising personal and family health/medical history, demographics, a physical and neuropsychological examination with a clinical assessor, questions about quality of life, daily living, physical activity, and measures of mood. The clinical and neuropsychological examination is repeated at yearly follow-up visits, as described in the following section.

#### Annual Clinical and Neuropsychological Examinations for Cognitive Status

MARS participants undergo an annual structured clinical evaluation, including a comprehensive neuropsychological battery of cognitive tests [30], such as the Mini-Mental State Examination (MMSE) [32], which is a commonly used standardized cognitive screening test with a maximum score of 30, where scores of 23 or lower indicate possible cognitive impairment. Clinical diagnosis of participant cognitive status is determined through a 3-step diagnosis process: computer scoring of cognitive tests, clinical review and judgment of data by a neuropsychologist blinded to participant demographics and previously collected data, and final diagnostic classification by a clinician upon review of all relevant data and examination of study participants. In this study, each participant’s cognitive status, either no cognitive impairment (NCI) or a diagnosis of MCI, was based on their final diagnostic classification from the annual study visit occurring closest to, and prior to, the onset of Illinois’ COVID-19 stay-at-home order. Participant demographic characteristics presented in this study include age (in years), sex, education (in years), and cognitive status and MMSE score. Age was also determined based on the study visit nearest to, and prior to, Illinois’ COVID-19 stay-at-home order.

### Longitudinal Data Collection

#### Overview

In addition to the clinical evaluation, continuous data were collected using the ORCATECH-CART technology platform, which includes (1) weekly online surveys to determine changes in behavioral, social, and physical patterns; and (2) actigraphy watch data to determine changes in physical activity and sleep patterns. A more in-depth description of the ORCATECH-CART technology platform has previously been published [27,28].

#### Weekly Health Update Surveys

Study participants are emailed weekly on Mondays with online surveys automatically via the Qualtrics Survey Platform (Qualtrics). If a survey is not completed, an automatic reminder email is sent on Wednesday, and if the participant does not respond after the reminder, follow-up phone calls are made to improve data capture. Completion of the full survey takes about 5 minutes, and participants can use any internet-capable device (eg, computer, tablet, or smartphone) to complete the survey [14,28,33,34]. The weekly survey includes 13 items (Table 1), which were designed to augment the behavioral data collected by the ORCATECH-CART platform with additional follow-up questions. Eleven of the 13 survey items require a yes/no response for a set of metrics regarding health, behavioral, and social activity patterns, such as away from home overnight, overnight visitors, blue mood, loneliness, medication changes, falls, accidents, hospitalizations, health limitations, living space change, and assistance change. The last 2 questions ask about pain levels and pain interference. Survey metadata are also collected, including submission date and timestamps, submission status (finished vs unfinished), and completion time. Data are stored on the Qualtrics servers and are accessed via application programming interface calls for data analysis.

**Table 1:** The 13-item weekly health update survey.

Question	Phrase	Response type
In the past week, have you been away from home overnight?	Away from home overnight	Yes/no
Have you had visitors who stayed with you in your home for a night or more?	Overnight visitors	Yes/no
Have you felt downhearted or blue for three or more days in the past week?	Blue mood	Yes/no
Have you felt lonely?	Loneliness	Yes/no
Have you had a change to any of your medications or started a new one?	Medication changes	Yes/no
Have you had a fall, including a slip or trip, in which you lost your balance and landed on the floor/ground/lower level?	Falls	Yes/no
Have you had any other injuries or accidents?	Accidents	Yes/no
Have you had any hospitalizations or emergency room visits (not including routine doctor visits)?	Hospitalizations	Yes/no
Has your physical health limited you more than usual: for example, did illness, pain, or arthritis keep you in bed or less active?	Health limitations	Yes/no
Have you had any changes in your home-space or living situation: for example, repairs, rearranged furniture, moved, someone moved in or left, or a new computer/cellphone?	Living space change	Yes/no
Is someone newly assisting you with medication management, bathing, dressing, or grooming?	Assistance change	Yes/no
Please rate your pain by indicating the number that best describes your pain on average in the last week.	Pain level	0-10
During the past week, how much did pain interfere with your normal activities or work (including both work outside the home and housework)?	Pain interference	1-5

#### Actigraphy Watch

For the collection of daily step count and sleep duration data, MARS participants were provided with and instructed to wear an actigraphy watch (Withings Activité; Withings) continuously, day and night, on their nondominant wrist. The Withings Activité was selected for its minimal technology demands. Its battery lasts approximately 8 months, eliminating the need for daily or weekly charging, and it enables passive data collection and monitoring without requiring users to press buttons or interact with the device. These low-effort features reduce user burden and are important for technology adoption among older adults [33,34].

### Data Analysis

#### Overview

Due to substantial differences in participant engagement across the data collection modalities and to ensure data quality and analytic validity, we defined 2 separate analytic subgroups for data analysis: 1 with available weekly survey data and 1 with available actigraphy watch data.

#### Weekly Survey Data Analysis

We examined weekly survey submission patterns among the MARS participants who were actively enrolled during the 32-week analysis window (November 30, 2019, to July 11, 2020) surrounding the onset of the Illinois COVID-19 stay-at-home order (March 21, 2020). Survey engagement was assessed across two 16-week periods pre– and post–COVID-19 based on weekly submission counts. The 16-week windows before and after the COVID-19 stay-at-home order were chosen to capture the acute impact of the Illinois stay-at-home order on participants’ survey engagement, behaviors, and wearable-derived activity, not to characterize long-term, seasonal, or chronic postpandemic trends. Each participant’s weekly survey submission status was classified into 1 of 3 categories: finished (when the participant began and submitted a survey), unfinished (when the participant began but did not submit a survey), or no survey (when the participant did not initiate a survey that week). The classification of unfinished vs no survey was used to distinguish partial engagement, as participants may have started but not submitted surveys for various reasons. In this study, this distinction was made for descriptive purposes and was not analyzed separately. However, prior research suggests that as individuals progress from NCI toward MCI, they may initially begin surveys but fail to complete them and, over time, stop initiating surveys, reflecting a gradual decline in engagement [35,36].

Participants were categorized by overall survey engagement. The “high-engagement” group was defined as submitting 50% or more of weekly surveys during the 32-week period, while the “low-engagement” group was defined as submitting fewer than 50%. The ≥50% engagement threshold was a prespecified pragmatic criterion, which is more conservative than completion cutoffs commonly used for ecological momentary assessment (EMA) study inclusion in similar populations [37]. The primary analyses of survey outcomes included all 59 participants under a missing-at-random assumption (see the Statistical Analysis and Missing Data subsections), while engagement status was retained as a sensitivity stratum. Survey start-hour time and survey completion-time analyses were conducted among high-engagement participants only, due to the necessity of sufficient submitted surveys per participant.

#### 13-Item Weekly Questions and Responses

From each participant’s submitted weekly surveys, we extracted responses to the 11 binary (yes/no) questions. Response frequencies were summarized weekly across participants for the full analytic cohort. Pre– and post–COVID-19 periods were analyzed separately to examine changes in response trends over time. The 2 pain-rating items (pain intensity on a 1-5 scale and pain interference on a 0-10 scale) were collected as part of the weekly survey but were not analyzed in this study.

#### Weekly Survey Start-Hour Time

To explore potential shifts in daily engagement behavior before and after the COVID-19 stay-at-home order, we analyzed the timing of survey initiation. Changes in survey start-hour patterns may reflect alterations in participants’ daily routines or availability, offering indirect insight into lifestyle disruptions. For each submitted survey, we extracted start times (recorded in hour:minute format) and binned them by hour for an analysis over a 24-hour period. To assess patterns in survey initiation, we computed the distribution of start times and quantified central tendencies using circular statistics appropriate for cyclical time data. Specifically, we calculated the circular mean and SD of survey start times during the pre– and post–COVID-19 periods. The circular mean hour was used to represent the central tendency of engagement time within the 24-hour cycle, and peak start hours were identified as the most frequently occurring hourly bins. All analyses were conducted separately for the pre– and post–COVID-19 time windows.

#### 13-Item Weekly Survey Completion Time

Additionally, to explore potential changes in participant engagement with the yes/no questions before and after the COVID-19 stay-at-home order, we examined completion time specifically for the 13-item weekly questionnaire. Differences in completion time were examined descriptively, as changes may reflect a range of possible factors, such as cognitive load, emotional burden, shifts in focus and attention, familiarity with the survey interface, or altered daily routines, though the specific underlying causes could not be determined. For each submitted weekly survey, we extracted the total time required to complete the 13-item questionnaire, which was defined as the elapsed duration between the 13-item questionnaire start and finish timestamps. Weekly completion times were averaged across participants and summarized using means and SDs for both the pre– and post–COVID-19 periods. These metrics were computed at the group level. To assess whether completion times differed significantly between the pre– and post–COVID-19 periods, we first applied the Shapiro-Wilk test to evaluate the normality of the data distribution. Depending on the result, we used either the Welch *t* test (for normally distributed data) or the Mann-Whitney *U* test (for nonnormally distributed data) to compare group-level completion times pre– and post–COVID-19. Given the small sample size, we anticipated deviations from normality, and distributions were also graphically inspected.

#### Actigraphy Watch Data Analysis

We analyzed actigraphy data over a 10-week window (February 15, 2020, to April 15, 2020) comprising 5 weeks before and 5 weeks after the Illinois stay-at-home order. This shorter window, relative to the 32-week survey window, was necessitated by reduced data availability stemming from COVID-19 lockdown disruptions to in-home technical support and device shipping, along with individual variability in watch wear. Participants were included in the data analysis if they had at least 5 valid days before and after the stay-at-home order, and days with implausible values (sleep durations >15 hours or step counts <50 per day) were excluded as likely nonwear or device error, as these thresholds fall well outside the plausible physiological range for community-dwelling older adults (mean total sleep time approximately 7 hours, with long sleepers conventionally defined as those sleeping more than 9 hours [38]) and are more lenient than the ≥100-step “valid day” floor used in actigraphy validation work [39]. For all included participants, daily step counts and sleep durations were aggregated separately for the pre– and post–COVID-19 periods.

#### Statistical Analysis

Baseline demographic and cognitive characteristics were summarized for the full analytic sample (N=59) and stratified by weekly survey engagement status (high- vs low-engagement). Across all analyses, continuous variables (eg, age, education, MMSE, daily step counts, and nightly sleep duration) were summarized using means and SDs, and categorical variables (eg, sex and cognitive diagnosis) were summarized using counts and percentages, computed at the participant level or aggregated across participants/weeks as appropriate. Between-group comparisons were conducted using Student *t* tests for continuous variables and chi-square tests for categorical variables.

#### Missing Data

The missingness mechanism for weekly survey responses was assessed prior to modeling. The Little test rejected the missing-completely-at-random hypothesis at a monthly aggregation (*P*=.002), and the test was not computable on the full 32-week response matrix owing to its high-dimensional sparsity, a known limitation of the Little test for intensive longitudinal data. Nonresponse was associated with baseline cognitive status (logistic regression odds ratio [OR] 0.18, 95% CI 0.03-0.89; *P*=.04; Fisher exact *P*=.03; 11/51, 22% of NCI vs 5/8, 63% of MCI participants were low-engagement), consistent with a missing-at-random (MAR) mechanism. We further examined whether COVID-19 onset precipitated complete survey cessation: exactly 1 continuously enrolled participant responded before but submitted no surveys after the stay-at-home order, and mean within-person response rates (each participant’s completed surveys out of 16 possible weeks per period, averaged across participants) were essentially unchanged (65.5% to 64.8%; 65.3% to 65.9% excluding that participant). Watch-data missingness was not formally assessed; its qualitative causes are described in the Actigraphy Watch Data Analysis subsection and the Limitations.

#### Primary Analyses

Generalized linear mixed models (GLMMs) with a logit link, a participant-level random intercept, and a binary pre– vs post–COVID-19 indicator (Illinois stay-at-home order, March 21, 2020) as the primary predictor, adjusting for age, sex, and education, were used to examine changes in each of the 11 binary survey outcomes. The primary analysis used the full analytic sample (N=59) under an MAR assumption, with high-engagement (n=43) and low-engagement (n=16) strata reported in parallel as sensitivity analyses. Within-participant correlation among repeated weekly observations was captured by the random-effects structure: weekly responses showed modest within-participant autocorrelation (mean lag-1 *r*=0.10) and substantial between-person variance (intraclass correlation coefficient–like estimate=0.39), both accommodated by the random intercept. Results are reported as ORs with 95% CrIs.

For continuous outcomes (daily step count and nightly sleep duration), linear mixed models with a participant-level random intercept were used to evaluate pre– vs post–COVID-19 changes, adjusting for age, sex, and education. Models were estimated using the PROC MIXED procedure in SAS (version 9.4; SAS Institute [40]). Results were expressed as coefficient estimates and SEs.

#### Sensitivity Analyses

To assess robustness of the survey findings under MAR, we conducted 3 sensitivity analyses: multiple imputation (20 imputations, Rubin rules), a single-case analysis excluding the 1 post–COVID-19 nonresponder, and an engagement-stratified analysis comparing the high-engagement (n=43) and low-engagement (n=16) groups separately.

Data analysis and visualizations were conducted using Python (version 3.9), and PROC MIXED and GLIMMIX models were performed using SAS software, with statistical significance evaluated at an α level of .05 for frequentist analyses and 95% CrIs excluding 1 for Bayesian estimates. Additional descriptive and statistical results are presented in Tables S1-S6 in Multimedia Appendix 1. In addition, software concordance checks comparing Python GLMM and SAS GLIMMIX implementations on the high-engagement subsample (n=43) are presented in Tables S3 and S4 in Multimedia Appendix 1.

## Results

### Weekly Survey

#### Participant Characteristics

A total of 59 MARS participants were active during the study period (Table 2). Of these, 43 participants (73%) were classified as the high-engagement group, defined as completing ≥50% of weekly surveys, and 16 participants (27%) were classified as the low-engagement group (<50% completion). Participants in the low-engagement group were significantly more likely to have been diagnosed with MCI (5/16, 31% vs 3/43, 7%; *P*=.03) and had lower mean MMSE scores (28.1, SD 1.4 vs 28.9, SD 1.0; *P*=.04) compared with participants in the high-engagement group. No significant differences were found between engagement groups with respect to age, sex, or years of education.

**Table 2:** Participant characteristics for weekly survey data analysis during the 16-week period before and after March 21, 2020: overall and stratified by high-engagement vs low-engagement.

Variables		Total (N=59)	High-engagement (n=43)	Low-engagement (n=16)	*P* value
Age (years), mean (SD)		76.6 (6.1)	76.8 (6.1)	76.1 (6.1)	.69
Male, n (%)		11 (19)	8 (19)	3 (19)	>.99
Education (years), mean (SD)		16.3 (2.9)	16.0 (2.8)	17.1 (3.2)	.21
Cognitive diagnosis					
	NCI^a^, n (%)	51 (86)	40 (93)	11 (69)	.03
	MCI^b^, n (%)	8 (14)	3 (7)	5 (31)	N/A^c^
	MMSE^d^, mean (SD)	28.6 (1.2)	28.9 (1.2)	28.1 (1.3)	.04

^a^NCI: no cognitive impairment.

^b^MCI: mild cognitive impairment.

^c^N/A: not available.

^d^MMSE: Mini-Mental State Examination.

#### Weekly Survey Submission Trends

During the 32-week analysis window, 622 surveys were completed in the pre–COVID-19 period (mean 38.88, SD 3.44 per week) and 613 surveys were completed post–COVID-19 (mean 38.31, SD 4.77 per week), reflecting consistent overall participation across time periods. When stratified by engagement status, the high-engagement group maintained stable engagement with 555 surveys completed pre–COVID-19 (mean 34.69, SD 2.89) and 566 surveys completed post–COVID-19 (mean 35.38, SD 4.53). Conversely, the low-engagement group demonstrated a decline in participation, completing 67 surveys before (mean 4.19, SD 1.87) and 47 surveys after COVID-19 onset (mean 2.94, SD 1.48), highlighting a reduction in weekly engagement during the pandemic period. Total and mean weekly submission counts before and after applying the ≥50% completion rate threshold are presented in Tables S1 and S2 in Multimedia Appendix 1.

**Figure 1:**
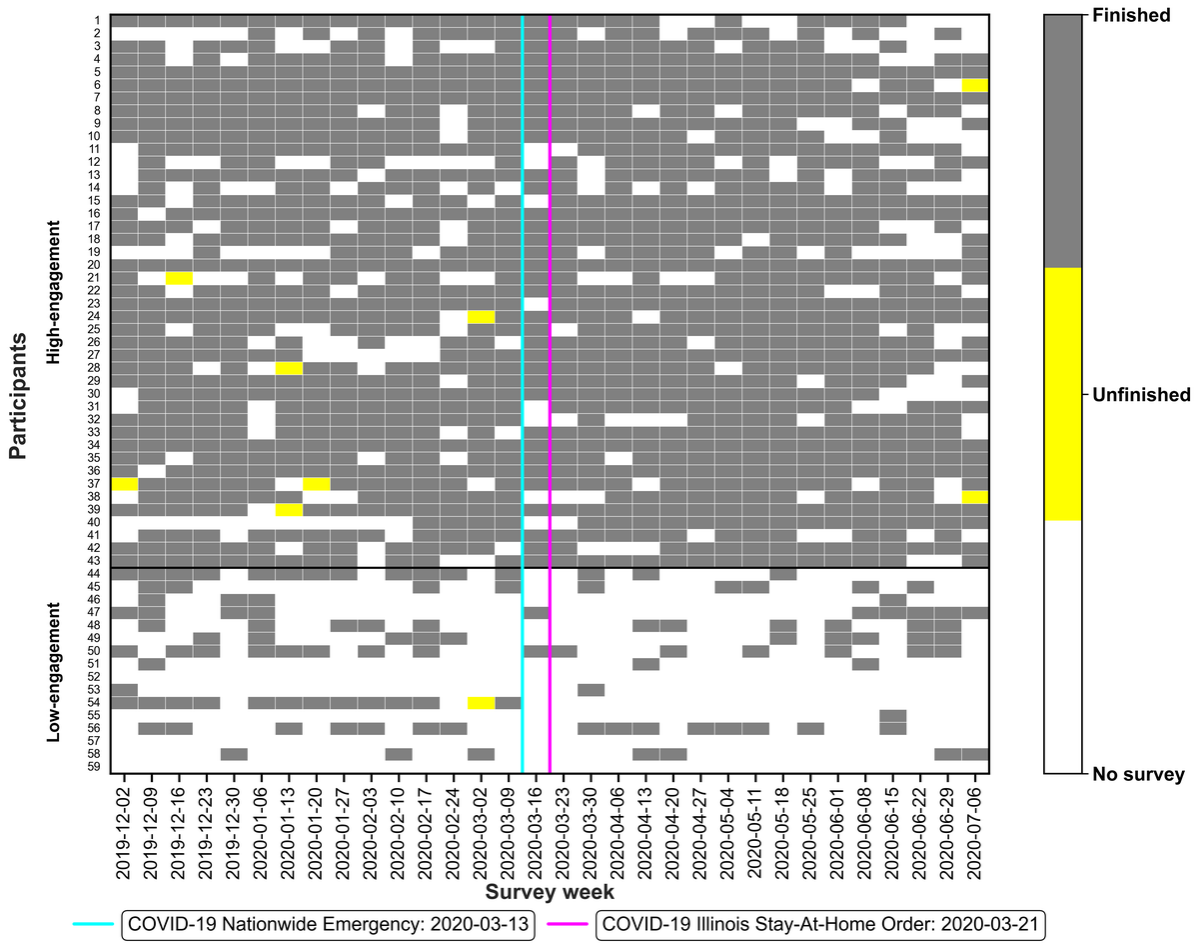
Heatmap showing individual-level weekly survey submissions across 16 weeks before and 16 weeks after the March 21, 2020, Illinois COVID-19 stay-at-home order. Survey status is color-coded: finished (gray), unfinished (yellow), and no survey (white). Participants are grouped by survey engagement: high-engagement, ≥50% of expected weekly surveys completed (n=43) vs low-engagement, <50% (n=16). The heatmap shows sustained submission among high-engagement participants and sparse, declining submission among low-engagement participants across the pandemic onset period.

**Figure 2:**
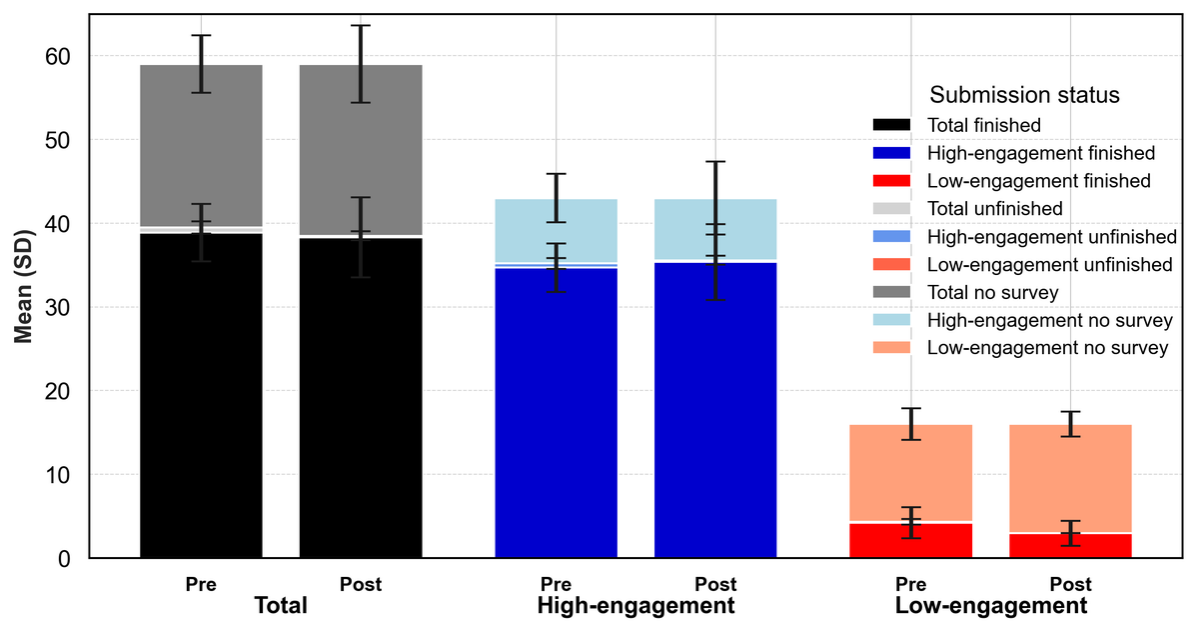
Stacked bar charts showing mean (SD) weekly survey counts by survey status type (finished, unfinished, and no survey) for the total, high-engagement, and low-engagement groups, comparing pre– and post–COVID-19 periods. Submission counts remained stable across periods for the total and high-engagement groups, while the low-engagement group showed reduced participation post-COVID-19.

Figures 1 and 2 visualize these trends, with Figure 1 presenting a heatmap of individual-level weekly survey submissions over the 32-week window, with submission status color-coded as finished (gray), unfinished (yellow), and no survey (white), and participants grouped by engagement classification (high-engagement, n=43; low-engagement, n=16). Figure 2 displays stacked bar charts of mean (SD) weekly survey submissions stratified by engagement group and time period. Each bar depicts the proportions of finished, unfinished, and no survey responses, highlighting the overall scarcity of participation among low-engagement individuals.

#### 13-Item Weekly Questions and Responses

Table 3 presents GLMM estimates of the odds of a “yes” response pre–COVID-19 vs post–COVID-19 for all 11 binary survey items, reported for the full analytic sample (N=59) and, in parallel, as sensitivity analyses, for the high-engagement (n=43) and low-engagement (n=16) subsamples. In the full sample, participants were significantly less likely after the stay-at-home order to report being away from home overnight (OR 0.30, 95% CrI 0.20-0.46), having overnight visitors (OR 0.44, 95% CrI 0.31-0.64), a medication change (OR 0.60, 95% CrI 0.42-0.86), or a health-related activity limitation (OR 0.70, 95% CrI 0.52-0.95). Reports of changes to the living space increased modestly (OR 1.41, 95% CrI 1.02-1.95). These 4 reductions were consistent in direction and magnitude in the high-engagement subsample. Estimates in the low-engagement subset (n=16) were unstable, being based on a few outcome-positive participants, and those that were nominally significant were attributable to a small number of repeat responders rather than group-level change. This instability is consistent with the missing-data assessment and is why the full-sample model, valid under an MAR assumption, is the primary analysis. Emotional well-being indicators (blue mood and loneliness) showed no significant change in the full sample. Estimates for rare events (accidents and new assistance needs) were unstable owing to very few responses and are reported descriptively only, and accidents were reported only during the pre–COVID-19 period. Age, sex, and education were included as covariates and were not the focus of inference.

Participants were significantly less likely to report being away from home overnight (OR 0.29, 95% CI 0.17-0.50; *P*<.001) or having overnight visitors (OR 0.52, 95% CI 0.32-0.86; *P*=.01) in the post–COVID-19 period compared with the pre–COVID-19 period. The covariates age at baseline, sex, and education were not significant predictors in any of the models. While pre–COVID-19 and post–COVID-19 differences were not statistically significant for other self-reported experiences (Table S3 in Multimedia Appendix 1), descriptive trends (Table S4 in Multimedia Appendix 1) revealed decreases in the group-level prevalence of medication changes, falls, and hospitalizations following the onset of COVID-19. Reports of accidents were rare and occurred only during the pre–COVID-19 period. Physical health limitations remained stable, while small increases were noted in changes to living spaces. Assistance needs remained minimal across both time periods. Table 3 presents GLMM estimates of the odds of a “yes” response pre–COVID-19 vs post–COVID-19 for all 11 binary survey items, reported for the full analytic sample (N=59) and, in parallel, as sensitivity analyses, for the high-engagement (n=43) and low-engagement (n=16) subsamples.

**Table 3:** Generalized linear mixed models (GLMMs) examining changes in the 11 binary weekly survey items before vs after the March 21, 2020, Illinois COVID-19 stay-at-home order, reported for the full analytic sample (N=59) as the primary analysis and for the high-engagement (n=43) and low-engagement (n=16) subsamples as sensitivity strata. Each row reports the post–COVID-19 effect (binary pre/post indicator) from a separate GLMM (with a logit link and a participant random intercept), adjusted for age, sex, and education; covariate estimates are not tabulated. Estimate: posterior mean (log-odds).

Survey item	Total (N=59)			High-engagement group (n=43)				Low-engagement group (n=16)		
	Estimate	SD	OR^a^ (95% CrI^b^)	Estimate	SD	OR (95% CrI)	Estimate		SD	OR (95% CrI)
Away from home overnight	–1.20	0.22	0.30 (0.20-0.46)^c^	–1.19	0.22	0.30 (0.20-0.47)^c^	–1.44		0.84	0.24 (0.05-1.24)^d^
Overnight visitors	–0.82	0.19	0.44 (0.31-0.64)^c^	–0.75	0.20	0.47 (0.32-0.70)^c^	–1.42		0.58	0.24 (0.08-0.76)^c,d^
Blue mood	0.00	0.27	1.00 (0.60-1.69)	–0.23	0.30	0.79 (0.44-1.42)	2.12		0.74	8.31 (1.96-35.14)^c,d^
Loneliness	0.40	0.24	1.49 (0.92-2.41)	0.20	0.27	1.22 (0.72-2.06)	1.25		0.65	3.49 (0.97-12.53)^d^
Medication changes	–0.50	0.18	0.60 (0.42-0.86)^c^	–0.39	0.18	0.68 (0.47-0.97)^c^	–1.36		0.69	0.26 (0.07-1.00)^c,d^
Falls	–0.67	0.37	0.51 (0.25-1.06)	–0.63	0.37	0.53 (0.26-1.10)	–1.14		1.44	0.32 (0.02-5.35)^d^
Accidents	–2.68	1.05	0.07 (0.01-0.53)^c,d^	–2.53	1.07	0.08 (0.01-0.65)^c,d^	–1.31		1.40	0.27 (0.02-4.20)^d^
Hospitalizations	–0.32	0.32	0.73 (0.39-1.35)	–0.35	0.32	0.70 (0.38-1.30)	—^e^		—	NE^f^
Health limitations	–0.35	0.15	0.70 (0.52-0.95)^c^	–0.32	0.16	0.73 (0.53-0.99)^c^	–0.98		0.71	0.37 (0.09-1.50)^d^
Living space change	0.34	0.16	1.41 (1.02-1.95)^c^	0.42	0.17	1.52 (1.10-2.11)^c^	–1.15		0.93	0.32 (0.05-1.96)^d^
Assistance change	–1.31	0.82	0.27 (0.05-1.34)^d^	–1.11	0.84	0.33 (0.06-1.70)^d^	–1.14		1.44	0.32 (0.02-5.37)^d^

^a^OR: odds ratio.

^b^CrI: credible interval.

^c^95% CrI excludes 1.

^d^Estimate based on fewer than 3 outcome-positive participants on at least 1 side of the COVID-19 split; driven by a small number of repeat responders and not interpretable as a group-level effect.

^e^Not available.

^f^NE: not estimable.

#### Weekly Survey Start-Hour Time

**Figure 3:**
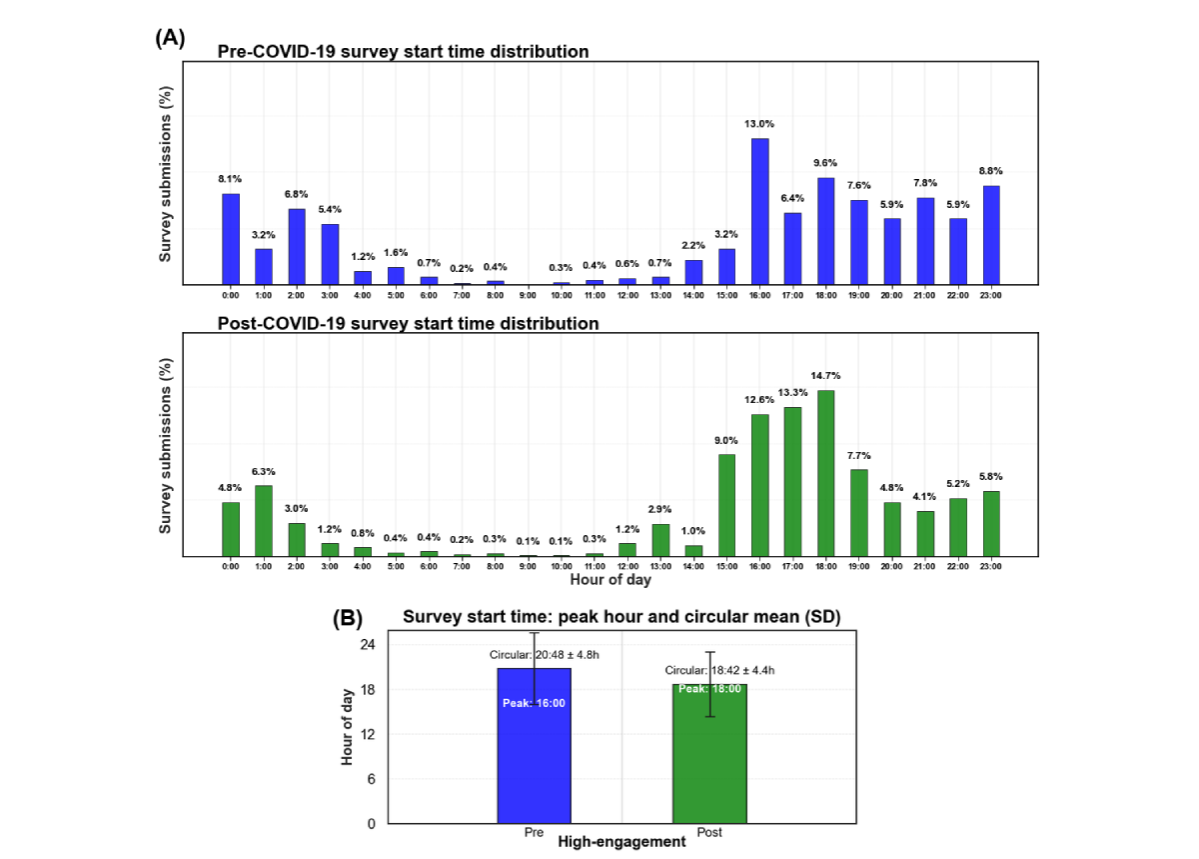
(A) Weekly survey start-hour time distribution over a 24-hour period comparing 16 weeks before and 16 weeks after March 21, 2020 (Illinois COVID-19 stay-at-home order) for the high-engagement group (n=43). (B) Group aggregate start-hour times computed using circular mean (SD) and peak hours for pre–COVID-19 vs post–COVID-19 for high-engagement participants. Bars represent circular mean (SD) survey start times, and peak submission hours are labeled at their respective y-axis levels. Post–COVID-19, the peak submission hour shifted later in the day, while the circular mean shifted earlier, and the distribution of start times became more tightly clustered.

Figure 3A displays the distribution of weekly survey start times over a 24-hour period for the 43 high-engagement participants, comparing the pre–COVID-19 and post–COVID-19 periods. Start times were summarized using 2 key metrics: the peak hour, representing the most frequently observed submission time, and the circular mean hour, capturing the central tendency of engagement within a cyclical 24-hour framework. In the pre–COVID-19 period, the peak submission hour was 16:00, shifting to 18:00 post–COVID-19, indicating a modest delay in preferred engagement time. The circular mean submission time shifted earlier by approximately 2 hours, from 20:48 pre–COVID-19 to 18:42 post–COVID-19 (Figure 3B), reflecting a broader trend toward earlier survey completion. The difference between the circular mean and the peak hour, used as an indicator of distributional spread, decreased from approximately 5 hours pre–COVID-19 to less than 1 hour post–COVID-19, suggesting a tighter clustering of engagement patterns over time.

#### Weekly Survey Completion Time

**Figure 4:**
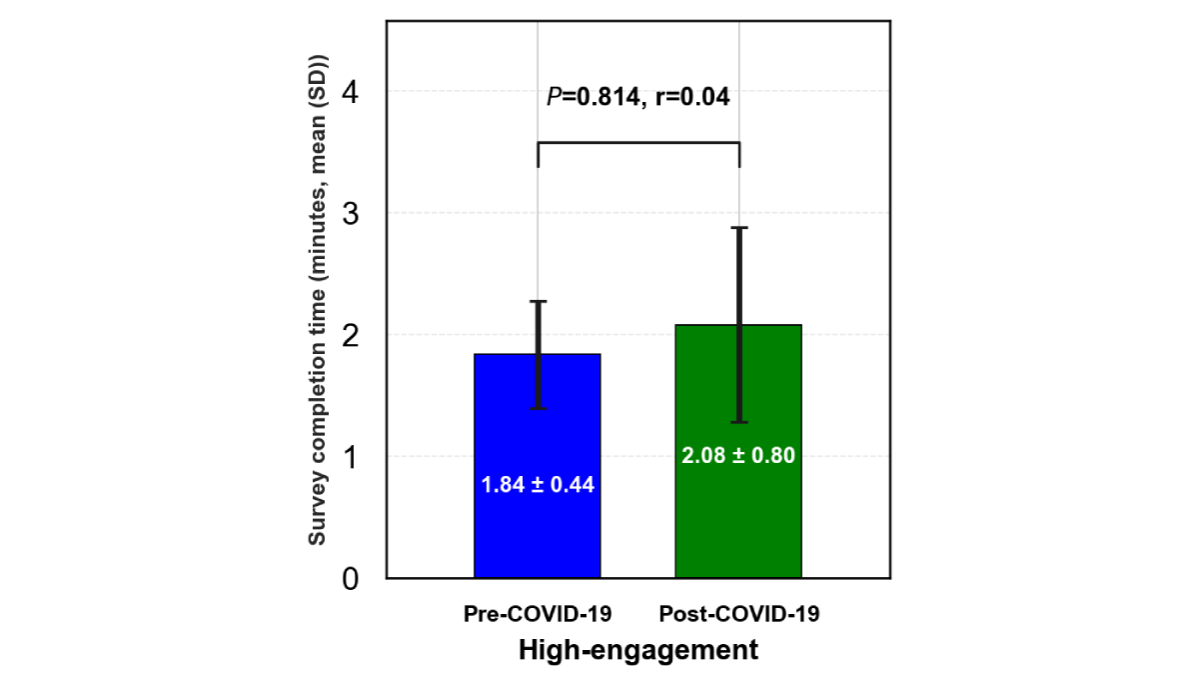
The 13-item survey completion time during the 16-week period before and the 16-week period after the March 21, 2020, Illinois COVID-19 stay-at-home order for the high-engagement group (n=43). Mean completion time increased slightly post–COVID-19 (from 1.84, SD 0.44 minutes to 2.08, SD 0.80 minutes), but the difference was not statistically significant.

Figure 4 displays the group-level mean (SD) completion times for the 13-item weekly survey, comparing pre–COVID-19 and post–COVID-19 periods among the 43 high-engagement participants. The average completion time increased from 1.84 (SD 0.44) minutes pre–COVID-19 to 2.08 (SD 0.80) minutes post–COVID-19. This difference was not statistically significant (*P*=.81), with a negligible effect size (*r*=0.04), indicating that the overall time required to complete the survey remained stable across the 2 periods.

### Wearable-Derived Daily Step Counts and Sleep Duration

**Figure 5:**
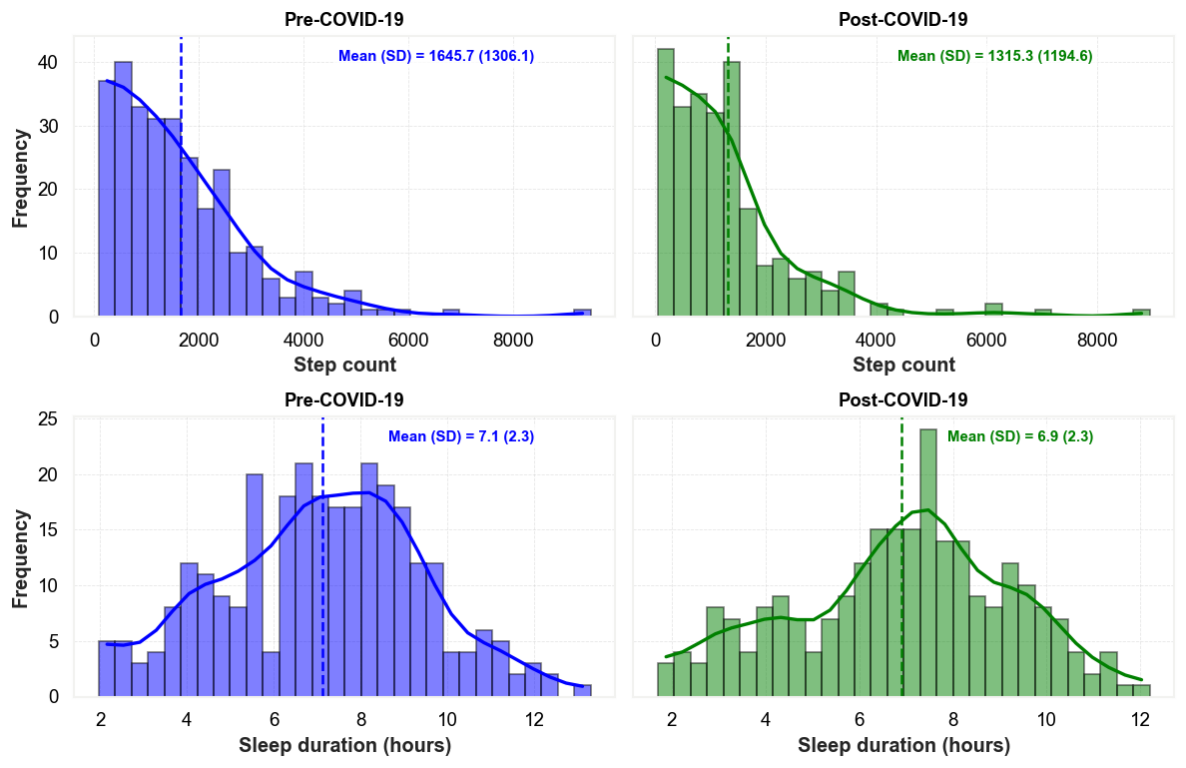
Distribution of daily step counts and sleep duration in the study analytic sample (n=15) during the 5-week period before and the 5-week period after the March 21, 2020, Illinois COVID-19 stay-at-home order. Daily step counts decreased significantly post–COVID-19 (from 1646, SD 1306 to 1315, SD 1195 steps; *P*<.001), while nightly sleep duration did not change significantly (7.1, SD 2.3 hours to 6.9, SD 2.3 hours; *P*=.49).

Table 4 describes characteristics of participants included in the data analysis based on valid actigraphy data (analytic sample, n=15), compared with those who were not included (n=44) due to insufficient or missing data. No significant differences were observed between the groups with respect to age, sex, years of education, cognitive status, or MMSE scores. Descriptive analyses indicated a reduction in daily step counts post–COVID-19, with mean (SD) steps decreasing from 1646 (SD 1306) steps pre–COVID-19 to 1315 (SD 1149) steps post–COVID-19. Similarly, nightly sleep duration declined from 7.1 (SD 2.3) hours to 6.9 (SD 2.3) hours post–COVID-19. Linear mixed models (Table 5) were used to formally test pre-post changes while adjusting for age, sex, and education, and confirmed a significant reduction in daily steps during the post–COVID-19 period (estimate –473.4, SE 66.1; *P*<.001). No significant associations were observed for age, sex, or education in relation to step counts. In contrast, the reduction in nightly sleep duration was not statistically significant (estimate –0.13, SE 0.19; *P*=.49). Age showed a positive association with nightly sleep (estimate 0.11, SE 0.07; *P*=.10). Sex and education were not significantly associated with sleep duration. Figure 5 displays the distribution of daily step counts and sleep duration.

**Table 4:** Participant characteristics for actigraphy watch data analysis during the 5-week period before and after the March 21, 2020, Illinois COVID-19 stay-at-home order. The analytic sample includes participants with at least 5 days of actigraphy watch use before and after March 21, 2020.

Variables		Analytic sample (n=15)	Not Included (n=44)	*P* value
Age (years), mean (SD)		75.9 (6.2)	76.8 (6.1)	.59
Male, n (%)		3 (20)	8 (18)	<.99
Education (years), mean (SD)		16.5 (2.6)	16.3 (3.0)	.82
Cognitive diagnosis				
	NCI^a^, n (%)	13 (87)	38 (86)	<.99
	MCI^b^, n (%)	2 (13)	6 (14)	N/A^c^
	MMSE^d^, mean (SD)	28.6 (1.0)	28.7 (1.3)	.79

^a^NCI: no cognitive impairment.

^b^MCI: mild cognitive impairment.

^c^N/A: not available.

^d^MMSE: Mini-Mental State Examination.

**Table 5:** Generalized linear mixed models (GLMMs) examining daily steps and nightly sleep duration measured by wearable devices before and after the March 21, 2020, Illinois COVID-19 stay-at-home order (n=15 participants).

Parameter		Estimate	SE	*P* value
Daily steps				
	Intercept	3261	2842	.27
	Post–COVID-19	–473.4	66.1	<.001
	Age	–1.2	35.3	.97
	Male vs female	–37.1	552.0	.95
	Education	–105.8	87.8	.23
Nightly sleep duration				
	Intercept	–3.60	5.40	.52
	Post–COVID-19	–0.13	0.19	.49
	Age	0.11	0.07	.10
	Male vs female	1.15	1.05	.27
	Education	0.10	0.17	.54

## Discussion

### Principal Findings

This study examined weekly health survey responses and actigraphy watch data before and after the onset of the COVID-19 pandemic among older African American adults enrolled in the longitudinal MARS study integrated with the ORCATECH-CART technology platform. We had four objectives: (1) assess overall survey engagement before and after the COVID-19 stay-at-home order; (2) analyze changes in behavioral, social, health, and wearable-derived activity patterns over the same period; (3) explore whether survey engagement was associated with cognitive status (post hoc); and (4) examine changes in participant interaction with the survey platform (start-hour timing and completion time, post hoc). Overall survey engagement remained stable across the pandemic onset period, although participants with low engagement (defined as <50% completion) were more likely to have MCI and lower MMSE scores. In the full analytic sample (N=59), GLMMs identified significant post–COVID-19 reductions in being away from home overnight, having overnight visitors, medication changes, and health-related activity limitations, while emotional well-being indicators (blue mood and loneliness) remained stable, and modest increases were noted in living space changes. Wearable data, available for a subset of 15 participants, showed a significant reduction in daily step counts with no significant change in nightly sleep duration. Start-hour timing of survey submissions shifted modestly post–COVID-19, while completion time remained stable.

### Comparison With Previous Research

The COVID-19 pandemic has been associated with widespread increases in social isolation and loneliness [2,10,41]. In a nationally representative study of 3257 US older adults (83% White and 6.5% Black), the pandemic was linked to increases in both outcomes, with 35% of respondents reporting loneliness during the outbreak, 22% reporting greater loneliness compared with preoutbreak levels, and 33% experiencing social isolation during the outbreak [2]. Although prior research [42,43] has shown that Black older adults are more likely to experience loneliness, Pica et al [2] found the opposite during the pandemic: Black older adults were more likely to be socially isolated but less likely to report loneliness than their White peers. Social isolation was defined as the absence or limitation of social contacts and interaction, including with family, friends, or group participation, whereas loneliness was assessed via self-reported experience.

In our sample, behavioral survey responses reflected expected pandemic-related lifestyle changes. Reports of being away from home overnight and receiving visitors declined significantly post–COVID-19, aligning with public health guidelines for physical distancing and stay-at-home compliance. These patterns were consistent with those observed in other cohorts using similar online health survey platforms. For instance, both Lennon et al [14] and Leese et al [12] documented significant reductions in travel and in-person social contact (eg, fewer overnight visitors) following the onset of the pandemic using the same weekly online health surveys and methodology in separate cohorts of older adults, including rural and urban veterans. However, in contrast to those studies that reported increased mood disturbance and loneliness [12,14], emotional well-being measures (low mood and loneliness) in our cohort remained low and stable across periods, suggesting relative psychological resilience among MARS participants who remained engaged with the study. Our findings add to growing evidence that, although opportunities for physical social interaction declined during the pandemic, many older adults were able to maintain emotional resilience and preserve a sense of connectedness through adaptive coping strategies [44].

Prior research has linked social isolation to increased fall risk and hospitalization [45], with reduced physical activity and limited social contact potentially increasing fear of falling [46]. In this study, descriptive trends showed numerical declines in falls and hospitalizations post–COVID-19, although these changes were not significant. Alongside the significant reductions in medication changes, these trends may reflect decreased mobility and physical activity due to stay-at-home orders, underreporting, reduced health care access, appointment cancellations, or hesitancy to seek care due to infection fears, particularly among older African Americans during the pandemic.

Despite the disruptions brought by the COVID-19 pandemic, overall survey engagement remained relatively stable in this study. The ≥50% engagement threshold used to define sensitivity strata was a prespecified pragmatic criterion that exceeds the approximately 33% completion benchmark used for EMA study inclusion, which a recent meta-analysis of EMA in cognitively diverse populations found was exceeded by all included cohorts [37]. While this threshold reflects reasonable engagement with study procedures, it may not necessarily reflect high digital engagement without direct measures of device activity. A reduction in survey engagement post–COVID-19 was observed in the low-engagement subgroup, suggesting that the pandemic may have widened preexisting engagement gaps among more vulnerable subgroups.

Analysis of survey timing revealed modest shifts in engagement patterns. Although the peak survey submission hour shifted to a later hour in the day, the circular mean survey start time moved earlier. Furthermore, the distribution of start times became more tightly clustered postpandemic. These findings suggest broader behavioral changes in daily routines during stay-at-home orders, potentially reflecting earlier structured activities or altered daily routines among older adults. While the mean survey completion time increased slightly post–COVID-19, the change was not statistically significant. These metrics of survey timing and duration have been shown to correlate with subtle cognitive decline in aging populations, as demonstrated in previous ORCATECH studies [27,36,47], which found that delays or variability in timing and duration of survey responses could signal emerging mild cognitive impairment. In this study, the relative stability of these indicators among high-engagement MARS participants suggests consistent interaction patterns with the survey platform through the early phase of the pandemic.

Objective wearable data revealed a significant decrease in daily step counts, while the reduction in nightly sleep duration was not statistically significant. Average step counts remained below the 25th percentile of age-matched norms (75-79 years) from the National Health and Nutrition Examination Survey 2005-2006, showing below-average physical activity levels both pre–COVID-19 and post–COVID-19 [48]. No significant associations were observed by age, sex, or education with either physical activity or sleep patterns, although a trend-level association between increasing age and greater sleep duration was noted. These findings are consistent with prior reports that physical activity levels in older adults declined during periods of restricted mobility [49,50]. Meanwhile, the relative stability of sleep duration in our sample may reflect potential resilience in maintaining basic sleep patterns, though the small sample size limits generalizability.

Disruptions related to COVID-19, such as limited in-person contact with research staff, difficulty resolving technical issues remotely, and reduced support from family and others, may have contributed to low engagement. These barriers were especially impactful among participants with cognitive impairment, who may require additional assistance to use and maintain digital devices. Other studies have documented similar challenges. For example, prior research [51] found that older adults reported difficulties using digital technology tools during the COVID-19 pandemic due to a set of factors, including unfamiliarity with technology, lack of targeted support, and fear of failure. These findings highlight the significance of providing clear instructions, individualized/personalized guidance, and ongoing support to ensure equitable digital participation, particularly for older adults with cognitive impairment.

### Limitations

Several limitations should be considered when interpreting these findings. First, to ensure data quality and consistency, we stratified participants by engagement, with low engagement defined as fewer than 50% completed weekly surveys before or after the pandemic onset. Participants with low engagement were more likely to have a diagnosis of MCI or lower MMSE scores, potentially introducing selection bias toward higher-functioning individuals within sub-analyses restricted to high-engagement participants (survey start-hour timing and completion time). The same cognitive status association indicates that data were not missing completely at random. We addressed this with a full-sample GLMM valid under a MAR assumption, together with multiple-imputation, single-case, and engagement-stratified sensitivity analyses, all of which supported consistent conclusions. However, item-level inference within the low-engagement subgroup was not statistically supportable given the small sample size and few outcome-positive participants per item.

Second, stable self-reports of emotional well-being should be interpreted cautiously. Participants who remained engaged with the study may represent a more resilient, socially connected, or digitally literate subgroup, and their stable reports may reflect a healthy responder or reporting bias rather than the absence of pandemic-related emotional impact. In addition, these items were captured as binary yes/no responses rather than continuous scales, which limits sensitivity to graded changes in mood and loneliness.

Third, the actigraphy sample was substantially smaller than the weekly survey sample. Although the Withings Activité watch was intentionally selected for its minimal technological demands, several factors may have contributed to lower engagement and data completeness: (1) disruptions in wearable data collection due to the COVID-19 pandemic, including limited in-person interactions, which made it difficult for the study team to resolve device issues in participants’ homes; (2) potential physical discomfort with wearing the watch continuously; (3) lack of perceived benefit in regularly wearing a watch; and (4) limited motivation to monitor personal activity or sleep among older adults.

### Conclusions

We explored a subset of older African American participants enrolled in the MARS study integrated with the ORCATECH-CART technology platform, examining digital engagement and health-related behaviors before and after the onset of COVID-19 stay-at-home orders in Chicago. The combination of weekly surveys and continuous wearable data enabled within-person comparisons across the pre- and postonset periods in a cohort that is underrepresented in both pandemic and aging research. The link between low survey engagement and MCI raises a broader concern for digital aging research: if participants with early cognitive decline are the most likely to disengage, digital tools risk capturing data primarily from the most engaged older adults rather than from the diverse populations they are intended to serve. Future research should prioritize strategies to support sustained engagement, mitigate technology-related barriers, and design digital research tools across multiple studies that account for cognitive variability in longitudinal studies of aging and health.

## Appendix

Multimedia Appendix 1 Supplementary survey submission summaries, generalized linear mixed model implementations, statistical analyses, prevalence comparisons, and sensitivity analyses.

## Abbreviations

CART: Collaborative Aging Research Using Technology
CrI: credible interval
EMA: ecological momentary assessment
GLMM: generalized linear mixed model
IRB: institutional review board
MAR: missing-at-random
MARS: Minority Aging Research Study
MARS-CART: Minority Aging Research Study – Collaborative Aging Research Using Technology
MCI: mild cognitive impairment
MMSE: Mini-Mental State Examination
NCI: no cognitive impairment
OHSU: Oregon Health and Science University
OR: odds ratio
ORCATECH: Oregon Center for Aging and Technology

## Data Availability

The datasets generated for this study will be available from the corresponding authors upon reasonable requests, which must include a clear scientific rationale and may require data use agreements and institutional review approval to ensure participant confidentiality and compliance with ethical standards.
