# Supplementary material for: COVID-19 Impact on Health Behaviors and Survey Engagement in Older African Americans: Longitudinal Cohort Study
Source: JMIR Form Res. 2026 Aug 3;10:e79432. doi: 10.2196/79432 (PMC13482242; doi:10.2196/79432)
Supplement: Supplementary file 1 [file formative_v10i1e79432_app1.docx]

***Supplementary Materials for***

**COVID-19 Impact on Health Behaviors and Survey Engagement in Older African Americans: Longitudinal Cohort Study**

Selda Yildiz^1,2^, PhD; Nora Mattek^1,2,3^, MPH; Joel Steele^1,2,4^, PhD; Sarah Gothard^1,2,3,5^, MS;
Bryan D James^6,7^, PhD; Ana W Capuano^6,8,9^, PhD; Lisa L Barnes^6^, PhD; Jeffrey A Kaye^1,2,3^, MD;
Zachary T Beattie^1,2,3^, PhD

^1^Department of Neurology, Oregon Health & Science University, Portland, OR, United States

^2^Layton Aging & Alzheimer's Disease Center, Oregon Health & Science University, Portland, OR, United States

^3^Oregon Center for Aging & Technology (ORCATECH), Oregon Health & Science University, Portland, OR, United States

^4^Department of Indigenous Health, University of North Dakota, Grand Forks, ND, United States

^5^Department of Epidemiology, University of Washington, Seattle, WA, United States

^6^Rush Alzheimer's Disease Center, Rush University Medical Center, Chicago, IL, United States

^7^Department of Internal Medicine, Rush University Medical Center, Chicago, IL, United States
^8^Department of Neurology, University of Chicago, Chicago, IL, United States

^9^Department of Public Health Sciences, University of Chicago, Chicago, IL, United States

Corresponding Author:
Selda Yildiz, PhD

**This document includes:**

Supplementary text

Tables S1 to S6

| **Table S1.** Total and Mean Weekly Survey Submission Type Counts during the 16-week period before/after 03-21-2020 Illinois COVID-19 Stay-At-Home Order prior to filtering for ≥ 50 % completion rate | | | | | | | |
| --- | --- | --- | --- | --- | --- | --- | --- |
| **Survey Status** | | **Finished** | | **Unfinished** | | **No Survey** | |
| **Group** | **Period** | **Total** | **(Mean ± SD)** | **Total** | **(Mean ± SD)** | **Total** | **(Mean ± SD)** |
| **Total** | Pre | 622 (65.89%) | 38.88 ± 3.46 | 10 (1.06%) | 0.63 ± 0.77 | 312 (33.05%) | 19.50 ± 3.49 |
| **Total** | Post | 613 (64.96%) | 38.31± 4.23 | 3 (0.32%) | 0.19 ±0.54 | 328 (34.70%) | 20.50 ± 4.03 |

*Mean (SD) or N (%) as appropriate. SD: Standard Deviation.*

| **Table S2.** Total and Mean Weekly Survey Submission Type Counts during the 16-week period before/after 03-21-2020 Illinois COVID-19 Stay-At-Home Order after filtering for ≥ 50 % completion rate | | | | | |
| --- | --- | --- | --- | --- | --- |
| **Survey Status** | | **Finished** | | **No Survey** | |
| **Group** | **Period** | **Total** | **(Mean ± SD)** | **Total** | **(Mean ± SD)** |
| **Total** | Pre | 555 | 34.7 ± 2.9 | 133 | 8.3 ± 2.9 |
| **Total** | Post | 566 | 35.4 ± 4.4 | 122 | 7.6 ± 4.4 |

*Mean (SD) or N (%) as appropriate. SD: Standard Deviation.*

**Python and SAS implementations of the generalized linear mixed models (GLMMs)**

To ensure comparable results between the Python and SAS implementations of the generalized linear mixed model (GLMM, fit using SAS PROC GLIMMIX), we fit the same model in both environments on the high-engagement subsample (N = 43). Two items (away from home overnight and overnight visitors) were modeled in both software; estimates were essentially in concordance (away from home: OR 0.29 SAS vs. 0.30 Python; overnight visitors: OR 0.52 SAS vs. 0.47 Python), supporting consistency across implementations. See table S3 for SAS GLIMMIX and S4 Python GLMM

| **Table S3. SAS PROC GLIMMIX** - examining two survey items before and after 03-21-2020 Illinois COVID-19 stay-at-home order (based on 1194 weeks of survey data from high-engagement participants (N=43) | | | | |
| --- | --- | --- | --- | --- |
| **Survey Item** | **Estimate** | **SE** | **Odds ratio (95%) CI** | **p-value** |
| **Away from home overnight** |  |  |  |  |
| Intercept | 0.87 | 2.67 |  | 0.75 |
| Post-COVID-19 | -1.23 | 0.27 | 0.29 (0.17 - 0.50) | <0.0001*** |
| Age | -0.04 | 0.03 | 0.96 (0.89 - 1.02) | 0.19 |
| Male vs female | -0.0001 | 0.54 | 1.00 (0.35 - 2.90) | 1.00 |
| Education | 0.007 | 0.07 | 1.01 (0.88 - 1.16) | 0.92 |
| **Overnight visitors** |  |  |  |  |
| Intercept | -10.52 | 6.15 |  | 0.09 |
| Post-COVID-19 | -0.66 | 0.25 | 0.52 (0.32 - 0.86) | 0.01** |
| Age | 0.09 | 0.08 | 1.09 (0.94 - 1.27) | 0.27 |
| Male vs female | -1.11 | 1.26 | 0.33 (0.03 - 3.92) | 0.38 |
| Education | 0.09 | 0.16 | 1.10 (0.80 - 1.51) | 0.57 |
| **p<0.05, **p<0.01, ***p<0.0001* |  |  |  |  |

| **Table S4.** Python GLMM - examining behavior and mood from weekly health form reports before and after 03-21-2020 Illinois COVID-19 stay-at-home order (based on 1194 weeks of survey data from high-engagement participants (N=43) | | | |
| --- | --- | --- | --- |
| **Survey Item** | **Estimate** | **SD** | **OR (95% CrI)** |
| **Away from home overnight** | -1.19 | 0.22 | 0.30 (0.20-0.47)* |
| **Overnight visitors** | -0.75 | 0.20 | 0.47 (0.32-0.70)* |

** 95% CrI excludes 1.*

**Before vs After COVID-19 Survey YES Response (%) for High-Engagement Group (N=43)**

**Changes in the percentage of “Yes” responses to the 11-item survey binary (YES/NO) questions are summarized in Table S3, comparing pre- and post-COVID-19 periods among the 43 participants with high-engagement.** The table presents group-level averages of individual participant-level means. For each participant, we first calculated the percentage of “Yes” responses across weekly surveys within each period (pre- and post-COVID). We then computed the mean and standard deviation of these participant-level percentages, providing a group-level summary of individual response patterns. Several items showed marked reductions in reported frequency following the COVID-19 stay-at-home order. For example, group mean prevalence of being **away from home** declined from **6.9% (SD 19.6)** pre-COVID to **2.0% (SD 8.3)** post-COVID**. Similarly, reports of having** **visitors** declined modestly from **8.4% (SD 21.2)** to **6.6% (SD 17.0)**. In contrast, measures of **emotional well-being** remained relatively stable. The proportion reporting **“blue mood”** stayed low across periods (**2.9% pre-COVID vs. 2.6% post-COVID**), and **loneliness** remained largely unchanged (**3.5% vs. 3.9%**, respectively). Indicators of **medical events** demonstrated notable declines. Group mean prevalence of **medication changes** decreased from **9.4% (SD 24.4)** to **3.9% (SD 12.4)**, **falls** declined from **2.7% (SD 8.7)** to **0.7% (SD 3.6)**, and **hospitalizations** decreased from **2.9% (SD 9.5)** to **0.7% (SD 3.2)**. **Accidents** were rare and reported only during the pre-COVID period. In contrast, the prevalence of **health-related activity limitations** remained stable (**8.0% pre-COVID vs. 7.8% post-COVID**). Group mean prevalence of **changes to living spaces** showed a slight increase from **4.1%** to **5.4%,** while **changes in assistance needs** remained very low across both periods (**0.3% vs. 0.1%**).

| **Table S5.** Prevalence of health event endorsements during 16-week periods before and after 03-21-2020 Illinois COVID-19 stay-at-home order: (%) among high-engagement group (N=43). | | |
| --- | --- | --- |
| **Survey Item** | **Pre-COVID Mean (SD)** | **Post-COVID Mean (SD)** |
| **Away from home overnight** | 6.93 (19.58) | 1.96 (8.30) |
| **Overnight visitors** | 8.38 (21.18) | 6.57 (16.95) |
| **Blue mood** | 2.92 (9.05) | 2.58 (6.37) |
| **Loneliness** | 3.52 (14.43) | 3.88 (13.20) |
| **Medication changes** | 9.42 (24.42) | 3.87 (12.36) |
| **Falls** | 2.69 (8.72) | 0.66 (3.64) |
| **Accidents** | 0.48 (3.13) | 0.00 (0.00) |
| **Hospitalizations** | 2.94 (9.54) | 0.72 (3.21) |
| **Health limitations** | 7.98 (21.69) | 7.76 (18.42) |
| **Living space change** | 4.08 (14.69) | 5.44 (15.56) |
| **Assistance change** | 0.25 (1.59) | 0.10 (0.55) |

*Group grand mean (SD). SD: Standard Deviation.*

| **Table S6.** Sensitivity Analyses Summary | | | |
| --- | --- | --- | --- |
| **Survey item** | **Primary (Total N=59)**  **OR (95% CrI)** | **Multiple imputation**  **OR (95% CI)** | **Single-case excluded OR (95% CrI)** |
| **Away from home overnight** | 0.30 (0.20-0.46)* | 0.52 (0.373, 0.737)* | 0.30 (0.20,0.46)* |
| **Overnight visitors** | 0.44 (0.31-0.64)* | 0.41 (0.281, 0.591)* | 0.45 (0.31,0.65)* |
| **Medication changes** | 0.60 (0.42-0.86)* | 0.52 (0.350, 0.783)* | 0.64 (0.45,0.90)* |
| **Health limitations** | 0.70 (0.52-0.95)* | 0.55 (0.394, 0.762)* | 0.71 (0.53,0.97)* |

*Note: * = 95% credible interval excludes 1. Multiple imputation (MI): 20 imputations, Rubin’s rules. Single-case exclusion: one continuously enrolled participant with no post-COVID survey submissions. All four robust findings remain significant and directionally consistent across sensitivity analyses; away-from-home is attenuated under MI, consistent with regularization given the high proportion of imputed observations.*
